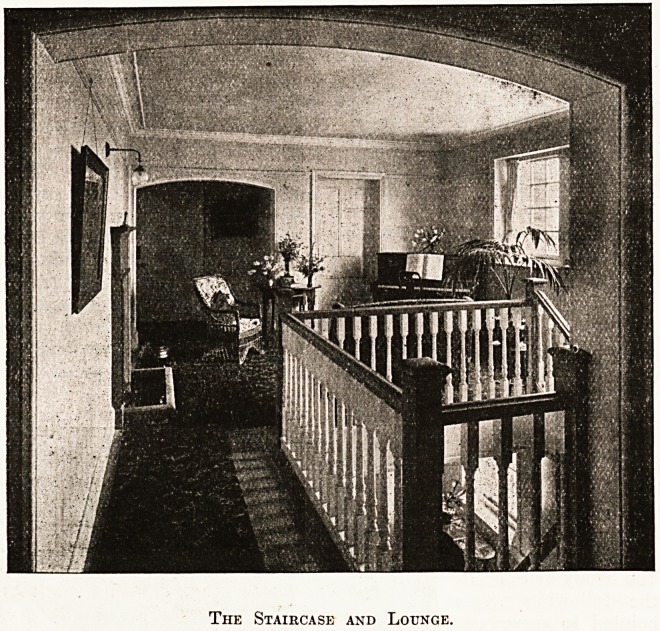# The New Research Hospital at Cambridge

**Published:** 1912-06-01

**Authors:** 


### THE NEW RESEARCH HOSPITAL AT CAMBRIDGE.

### Its Development, Opening, and Aims.

On Friday last week Mr. R. C. Brown, F.R.C.S., F.R.C.P., of Preston, formally opened, in the presence of a large and brilliant gathering, the New Research Hospital, which has been erected on the Hills Road overlooking the Gogmagog

The Front View of the Research Hospital.

Hills. The Vice-Chancellor, the President of the Royal College of Surgeons, and Sir William Osler were unable to be present. Sir Clifford Allbutt, K.C.B., Dr. Norman Moore (representing the Royal College of Physicians), Professor J. B. Bradbury, Professor Sims Woodhead, and Dr. Laurence Humphry were amongst those present.

This Research Hospital has emanated from the labours of those who have been engaged in the study of rheumatoid arthritis. Some five years ago steps were taken to form a Committee for the Study of Special Diseases, and the following were among those appointed to serve on the Committee: Sir Clifford Allbutt, K.C.B., Sir William Osler, Sir William Church, Sir Jonathan Hutchinson, Sir Victor Horsley, Sir Donald MacAlister, Sir Richard Douglas Powell, Sir Thomas Barlow, Sir Watson Cheyne, Sir Henry Morris, and Professor F. Howard Marsh. In addition to the Committee, the following were appointed honorary treasurers: Sir Clifford Allbutt, K.C.B., Regius Professor of Physic in the University of Cambridge; Sir W. Selby Church, Bart., K.C.B., Professor G. Sims Woodhead, and Dr. Strangeways, Lecturer in Special Pathology.

The work which this special hospital has set out to accomplish consists of giving relief to the individual patient, benefiting others suffering from similar diseases by throwing light on their origin and treatment, discovering means of preventing their onset in those at present healthy, and by imparting the knowledge thus gained to others. This is in itself a work of profound importance and significance.

Much and very fruitful research has been done in acute diseases, but there are still many chronic and widespread maladies which have not yet received the attention that they deserve. These maladies, although not immediately fatal to life, incapacitate the sufferer from active work, inflict great pain and misery, and finally leave him helpless and entirely dependent upon the kindness of his relatives and friends. Such cases cannot usually be admitted into our general hospitals, as they are chiefly for patients suffering from acute diseases for which some relief or cure can be afforded.

The diseases which the Committee had in mind had not at that time—five years ago—attracted the serious attention of the pathologist, in fact, not until quite recently; while to the physician these diseases were unsatisfactory as offering no field for brilliant efforts of curative skill. They were mere wastes of dulness, unrelieved by scientific interest, and in many cases uncheered by the prospect of effecting much good.

The Research Rooms and Museum.

The existence of a properly appointed and efficiently equipped Research Hospital will of itself serve to give a great impetus to the study of the diseases for the investigation of which it has been founded.

All those concerned in the founding of this valuable institution and the work upon which they have embarked are to be congratulated upon their persevering efforts and upon the results so far attained.

The elevations show that the Research Hospital has been designed with a view to combine simplicity with economy, and is of Georgian style. A striking feature is the provision of balconies, which will undoubtedly prove to be of service in the treatment of patients.

THE STAIRCASE AND LOUNGE.

On the ground floor is the entrance hall with fireplace and well staircase to the first-floor, consulting room and library, three research rooms, *x*-ray apparatus and dark-room, together with the kitchen, scullery, and other domestic offices. On the first floor is a spacious landing set out and furnished as a lounge for the patients, as our illustration shows, having wards opening directly off, which is most convenient for the patients, and on this floor access to two balconies is obtained from the central ward. The sisters' room is so arranged as to command full view of all the wards, of which there are four on this floor, with bath rooms, lavatories, and linen room. It is satisfactory to find that the sanitary block secures cross-ventilation. Another feature is the provision of a well-arranged broad spacious terrace overlooking the Gogmagog Hills from the south-east and south-west aspects.

The general construction is of such a nature as to admit of the building being used not only as a hospital, as it now is, but either as a private residence or as a hospital and laboratory combined should occasion arise.

The attention of the medical profession is drawn to the self-sacrificing labours of those who have been, and still are, devoting themselves to the study of a disease at once so obscure and uninteresting to the general practitioner as rheumatoid arthritis. So far, medicine has been able to effect but little relief to the sufferers. The culturation ground which the Cambridge workers are preparing will, it is hoped, provide a bountiful harvest. It may be that the pioneers in this task will share the fate of other early workers, and witness others reaping the fruit of their labours. However, it is with them a labour of love. But the most grateful mark of appreciation that could be accorded these pioneers would be sufficient support to enable the hospital to enter upon its career of usefulness free of debt. The Committee, it will be seen, consists of the most brilliant members of the medical profession, and the work already begun could not be under better auspices, and for the work to be hindered by lack of funds would be nothing less than a disaster to the public.

We are indebted to the courtesy of Dr. Strangeways for the photographs which accompany this description.

## Figures and Tables

**Figure f1:**
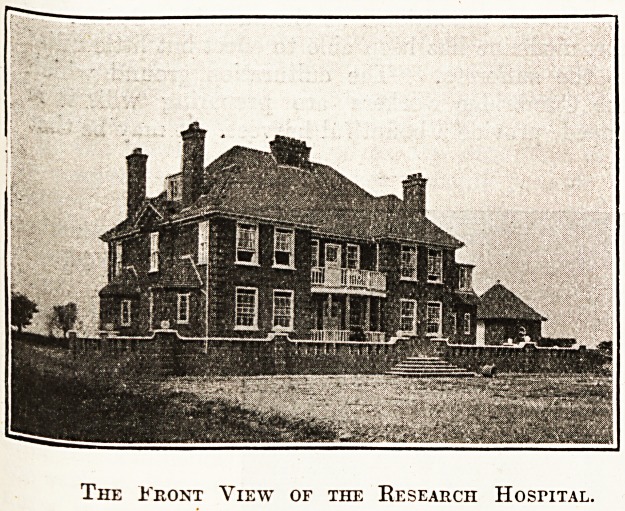


**Figure f2:**
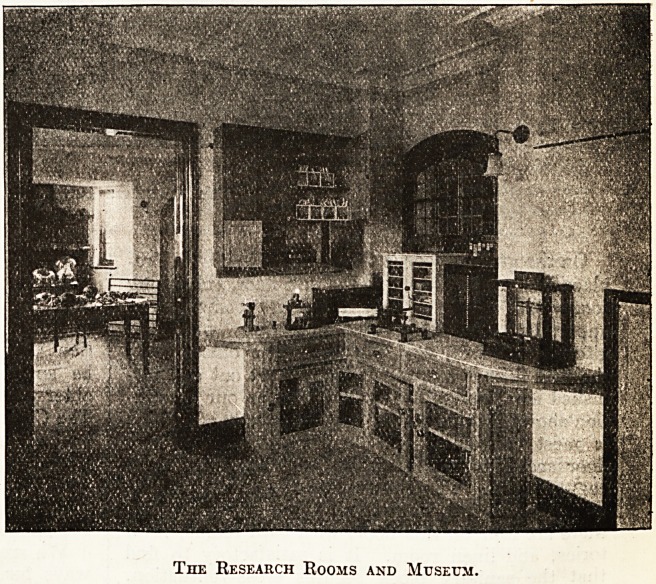


**Figure f3:**